# A Projection and Density Estimation Method for Knowledge Discovery

**DOI:** 10.1371/journal.pone.0044495

**Published:** 2012-10-01

**Authors:** Adam Stanski, Olaf Hellwich

**Affiliations:** Technical University Berlin, Computer Vision and Remote Sensing Group, Franklinstr. Berlin, Germany; Rutgers University, United States of America

## Abstract

A key ingredient to modern data analysis is probability density estimation. However, it is well known that the curse of dimensionality prevents a proper estimation of densities in high dimensions. The problem is typically circumvented by using a fixed set of assumptions about the data, e.g., by assuming partial independence of features, data on a manifold or a customized kernel. These fixed assumptions limit the applicability of a method. In this paper we propose a framework that uses a flexible set of assumptions instead. It allows to tailor a model to various problems by means of 1d-decompositions. The approach achieves a fast runtime and is not limited by the curse of dimensionality as all estimations are performed in 1d-space. The wide range of applications is demonstrated at two very different real world examples. The first is a data mining software that allows the fully automatic discovery of patterns. The software is publicly available for evaluation. As a second example an image segmentation method is realized. It achieves state of the art performance on a benchmark dataset although it uses only a fraction of the training data and very simple features.

## Introduction

Probability density estimation is arguably the most fundamental approach of learning from data. Theoretically, a density estimation could be used to answer the major questions arising in problems like regression, ranking, classification, clustering, feature selection, or outlier detection. For example, classification is reduced to asking for the highest probability of all classes and outlier detection translates to the questions for data points with low density. The answers could be given with ease based on an evaluation of a precise density estimation at various locations.

Unfortunately, in practice a density estimation, which is equally universal and precise, is out of reach due to the curse of dimensionality, see [1]. For a finite data set one is forced to include assumptions to estimate a precise density. However, by incorporating assumptions about the data, the estimator is no longer universal. An apparent example are parametric estimators. They use the assumption of a functional form of the density to simplify the estimation. Likewise, practical non-parametric estimators require assumptions about the data. This is illustrated with two examples in the following.

Vincent et al. [2] propose a modified kernel density estimator for manifolds. The underlying assumption is that a local fitting of kernels to their neighboring data points improves precision. On data, which is embedded in manifolds and therefore has a distinct local structure, an increased performance is demonstrated. A more application-specific example is given by Miller et al. [3]. Their goal is to estimate a density in a computer vision context. They calculate the probability of the appearance of an image with different transformations. This requires the estimation of a four-dimensional density of affine transformations. Their solution assumes that a newly proposed invariant distance function simplifies this task. Experimental results confirm the superiority over the simple Euclidian distance. Both methods are typical examples of how to overcome the curse of dimensionality: they use a fixed set of assumptions, namely a local kernel fitting and a specific distance function.

This paper contributes by following an alternative path to precise density estimation. Instead of a fixed set of assumptions we propose a framework that allows a flexible choice of assumptions. It supports the adjustment of assumptions to the specific task at hand, creating a tailor-made model. This is done by means of 1d-decomposition, which is the decomposition into one or multiple 1d-distributions. If a problem can be modeled as a 1d-decomposition, the framework allows a precise as well as fast computation of densities.

The outline of the remainder of this paper is as follows. The proposed method, called constructive probabilistic learning, is described in the methods section. Its application is demonstrated with a synthetic example in the subsequent section. The paper continues with two real-world examples that illustrate the wide range of possible applications for automatic data mining and image segmentation. Finally, the last section provides our conclusions.

**Figure 1:**
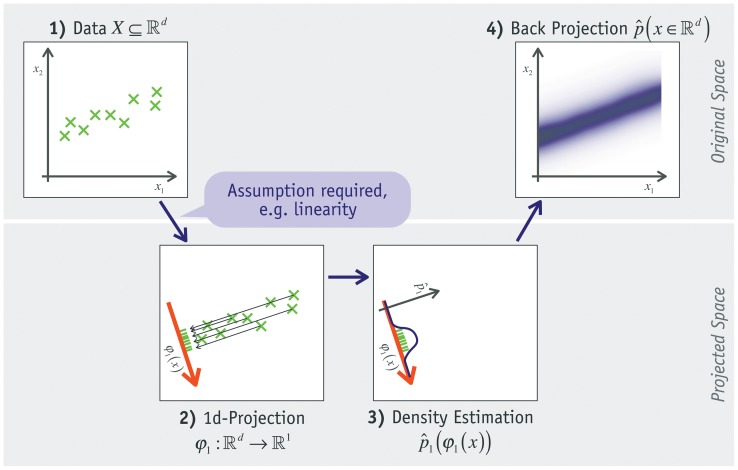
Basic idea of proposed method. The basic idea of the Cepel method using the example of a single linear projection.

## Methods

A probability density function 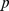 describes a distribution in a 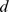-dimensional continuous space. It allows to calculate the probability 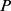 that a point 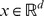, drawn from the distribution, occurs in volume 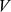 (see e.g. [4]):
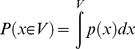
(1)


Calculating 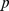 without further assumptions would require an infinite amount of data. In practice however, only a limited number of points 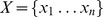 is given. Therefore, only an estimation 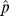 of the probability density function can be achieved. A well-defined solution is impossible, because 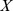 could have been drawn from any nonzero 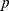. Accordingly, no nonzero estimation 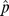 can be ruled out, although some are very unlikely.

**Figure 2:**
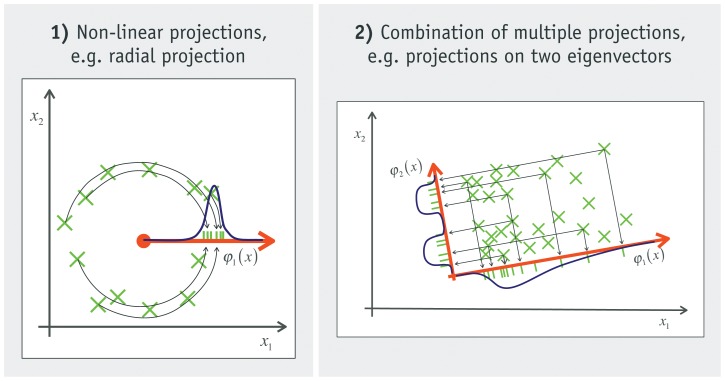
Two characteristics of basic idea. Projections can be non-linear and multiple projections can be combined.

Constructive probabilistic learning, or Cepel, is a method to perform this estimation. It is based on the idea of calculating all estimations in one dimension instead of in the original multidimensional space. For this purpose, the data is projected to 1d-spaces, in which a density estimation with high precision is possible. A Cepel model combines those densities back to an estimation in the original 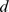-dimensional space. By deciding which projections to use and how to combine them, various assumptions about the data can be included. A Cepel model 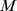 estimates a 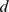-dimensional probability density 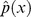 by combining the estimations of multiple 1d-projections created by functions 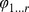. The Cepel model is defined by:
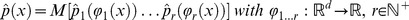
(2)


Figure 1 gives a simplified illustration of the idea in four diagrams: 1) The first shows the 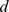-dimensional data 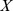 whose density is to be estimated. 2) Each data point is projected to one dimension (red arrow) by function 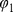 resulting in a 1d-distribution of the data. Assumptions about the data must be made to choose an appropriate projection function. 3) The probability density 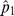 of this 1d-projected distribution is estimated. 4) This 1d-estimation is projected back into the original space. Here it can be normalized if required for the task at hand (assuming that the space is bounded). The result is a probability density estimation for each point in 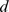-dimensional space of input data.

**Figure 3:**
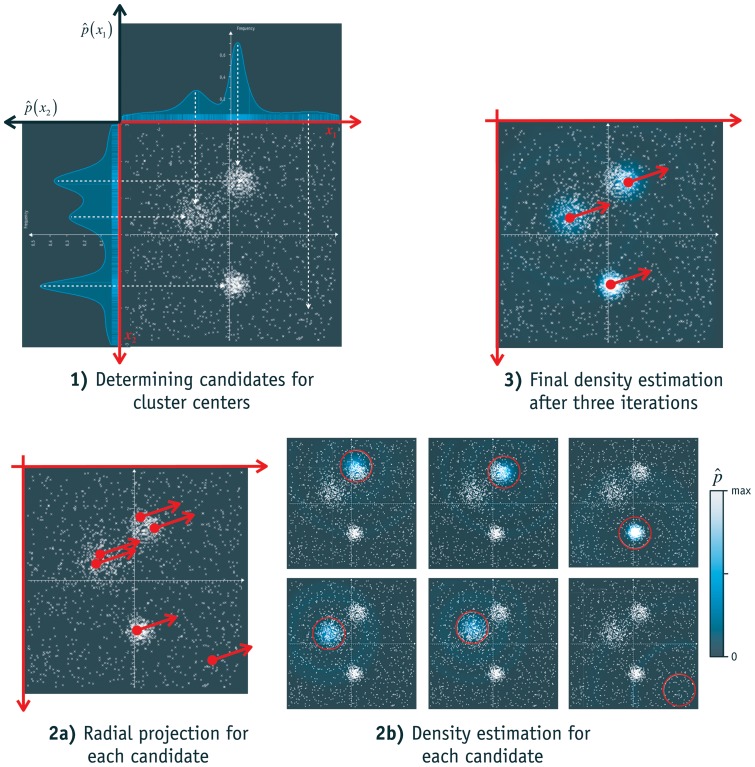
Example of density estimation of clustered data. The density of data with known properties is estimated in three steps. Only the first two dimensions of the multidimensional data are shown.

The Cepel method is more general than this simple illustration regarding two aspects, see figure 2. Firstly, projections are not restricted to linear functions. Any function that calculates a scalar value from a multidimensional vector is applicable. Secondly, multiple 1d-projections can be performed yielding various 1d-density estimations. They are combined to a 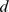-dimensional estimation using, e.g., a multiplicative, a conditional or a maximum operator. Examples of efficient ways of combination are given in the remainder of this paper.

**Figure 4:**
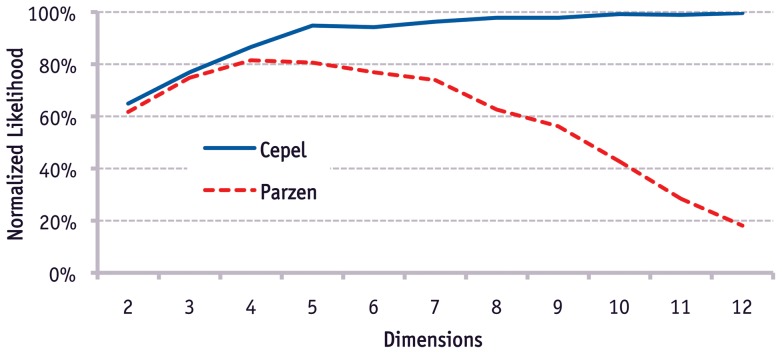
Evaluation of precision of estimation. Comparison of estimation with Parzen window and Cepel on clustered data as shown in figure 3.

**Table 1:** Equations of some of the models used.

Name	Equation
Single Axis	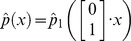 with vector 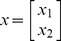
Naive Bayes	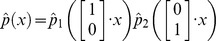
Regression	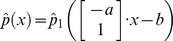 with regression line 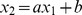
Radial	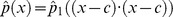 with center 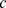
Eigenvectors	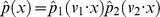 with eigenvectors 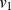 and 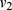
Logarithmic	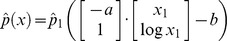 with regression 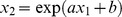

Equations of various standard methods converted to density estimations based on 1d-decompositions.

A crucial question is how to choose the projective function. Two approaches are possible. Either valid assumptions about the data are known that allow a 1d-decomposition. For this, the user has to understand exactly which features of the data are relevant for characterizing its distribution. This requires experience but allows to model a complex problem with highest precision. This direct modeling approach is exemplified in the introductory example and image segmentation section.

**Figure 5:**
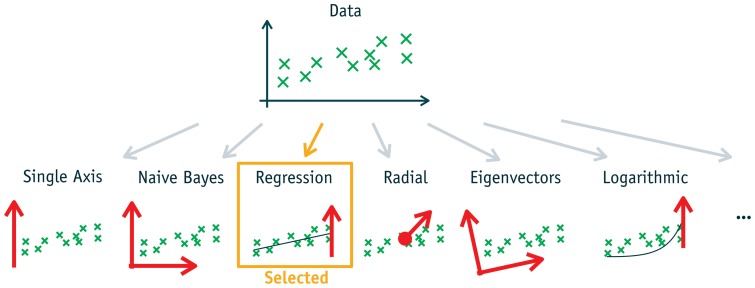
Automatic data mining approach. Automatic data mining by evaluating various 1d-decompositions and selecting the most likely. The decompositions correspond to the equations in table 1.

Alternatively, we use projective functions that are frequently applicable independent of the source of data. For example, linearity or logarithmic distributions appear commonly in nature. By applying various of these projective functions and selecting the most precise, we obtain a fully automatic modeling procedure. This approach is realized in the automatic data mining section.

**Figure 6:**
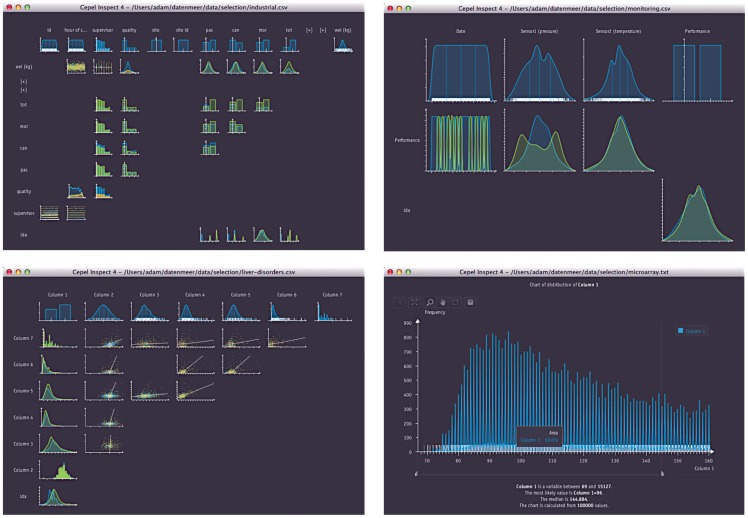
Screenshots of Cepel Inspect. Various publicly available data sets (from [7]–[8]) are analyzed with the software. The analysis creates a varying number of charts depending on the number of columns in the data and their explanatory power.

If the data was successfully decomposed into 1d-projections, no further assumptions are required to calculate a consistent density. As all estimations are performed in one dimension, the approach can handle very limited data with high precision. It is thereby not affected by the curse of dimensionality. It works equally efficient with very large scale data sets, because density estimation in one dimension provides many means for optimization. The method used for 1d-estimation is described in Section 1 of Appendix S1. It consists of an adaptive kernel density estimator whose bandwidth is selected with a likelihood criterion. A summary of strategies to reduce runtime is given in Section 2 of Appendix S1.

**Figure 7:**
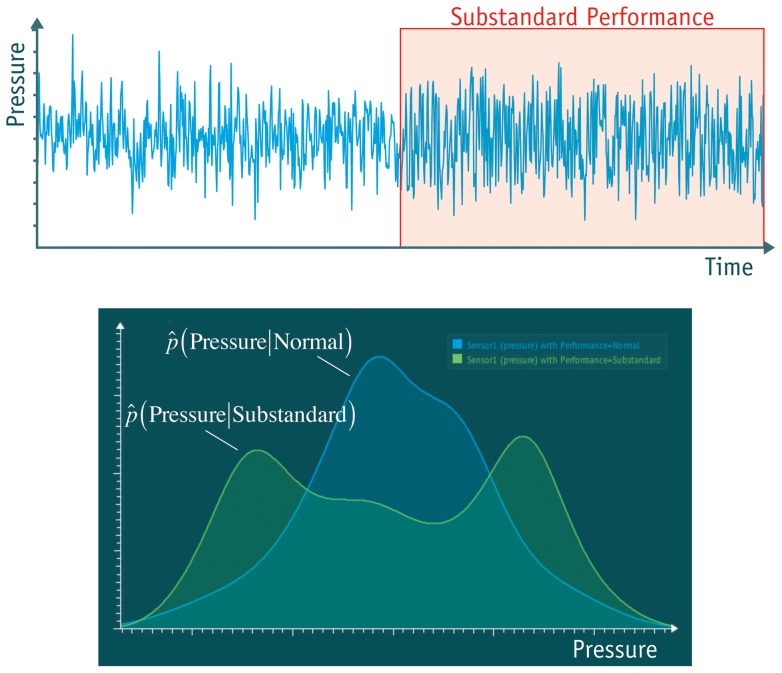
Example of a result created in the analysis. Upper diagram: the difference of the signal between the left and right half is easily missed. Lower diagram: the same data displayed as distributions. The blue curve is calculated from the left part of the signal; the right half is displayed in green. The difference between both distributions is considerable and can be detected automatically.

## Results

### Introductory Example

The following example illustrates how assumptions about a task are used to select appropriate projections and how to combine the 1d-density estimations based on them. The task is to estimate the density of data with some specific properties: the data is clustered, high-dimensional, and distorted by uniform noise. This knowledge about the problem is utilized to construct a tailor-made model based on 1d-projections. Figure 3 outlines the approach in three steps.

**Figure 8:**
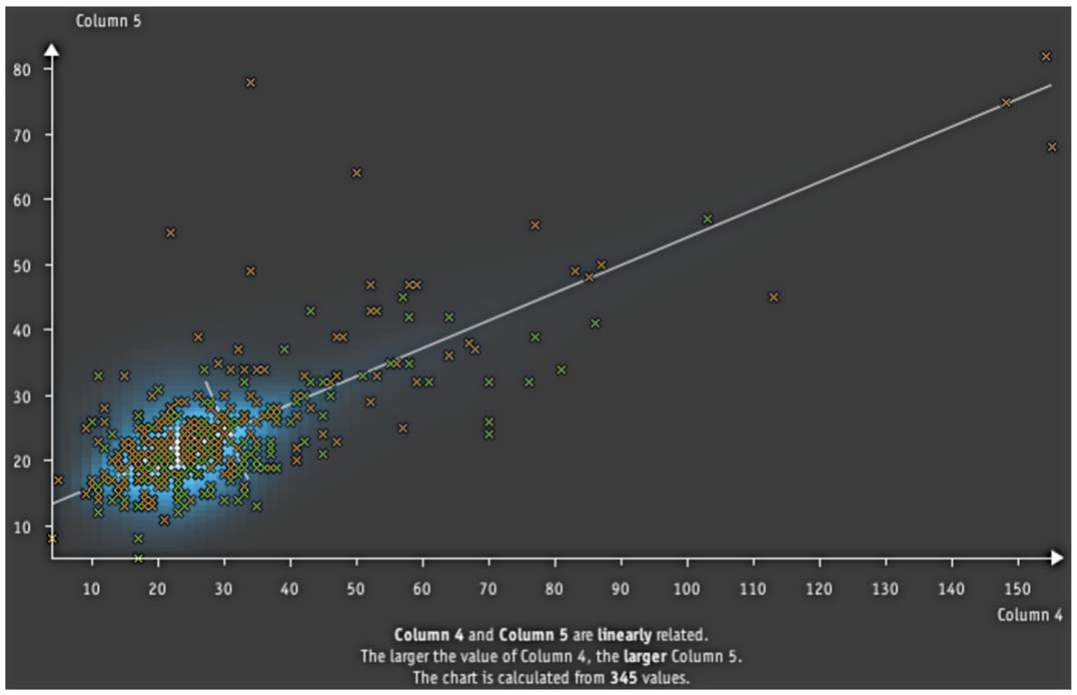
Another example from the analysis. The diagonal line indicates the linear relation that was automatically extracted from the data.

**Table 2:** Overview of features used in segmentation.

Feature	Posterior	Description
Brightness	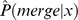	The difference of average grey values of two regions.
Texture	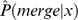	The difference of variance of grey values of two regions is used as a simple measure of texture. For example, a texture value of zero means that the variance of two regions is equal.
Arrangement	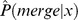	The arrangement of two regions relative to each other, calculated as a percentage depending on the number of pixels on their borders. It is calculated by dividing the number of pixels on outside borders (that touch only one of the two regions) by all pixels at borders (including pixels between the regions). For example, two nested regions have an arrangement value of zero percent.
Size	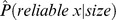	The size of a region, measured by the number of pixels it contains.

The features are required to calculate one of the posterior probabilities for ‘merge’ or ‘reliable’.

The goal of the first step, illustrated in diagram 1 of figure 3, is to find candidates for cluster centers. This is achieved by projecting the data onto each axis, performing a 1d-density estimation and calculating its maxima. The data points whose projected values are closest to a maximum are picked as a possible cluster center. In this example, three maxima are found on each axis. The 1d-spaces on which data is projected are marked with red arrows. The closest data points are denoted by a dashed arrows. Notice that these candidate points are just rough estimations, not optimal cluster centers. Some are not even inside of a clustered region like the rightmost point.

**Figure 9:**
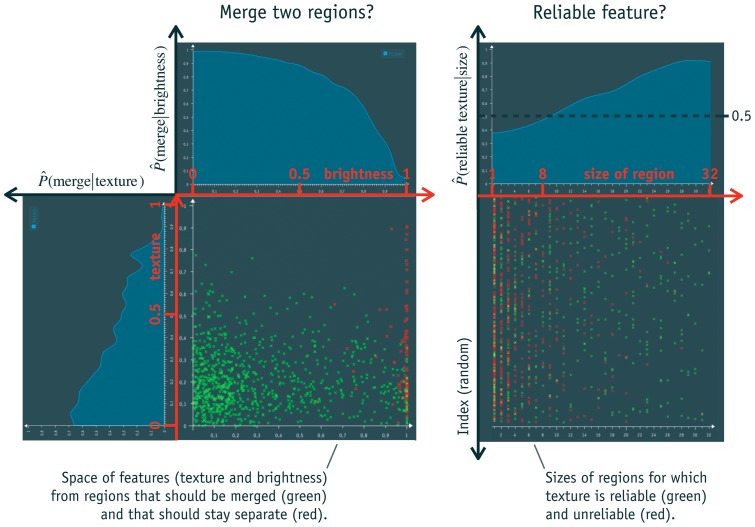
Decomposition of merging probability. The probability that two regions are merged is decomposed into 1d-distributions of feature values (left) and the reliability of each feature depending on size of the regions (right). The left diagram shows two out of three features; on the right, texture is used as one example out of the three features.

In the second step the candidates are used to simplify the density estimation. This is done with a radial projection for each candidate (as exemplified on the left of figure 2). To create a radial projection, the distances between a candidate and all data points are calculated. This leads to a 1d-distribution of distances for every candidate. The densities of these 1d-distributions are estimated and projected back into the original space. The result is a density estimation around each candidate as shown in diagram 2 b of figure 3. It can be seen that densities are only high (light blue) if a candidate is indeed a cluster center. For example, the rightmost candidate in diagram 2a is not inside a cluster. Thus, the corresponding radial estimation (last image in 2 b) does not contain a concentration of probability density. All other radial estimations show a high density in their center, because they originate in a cluster.

**Figure 10:**
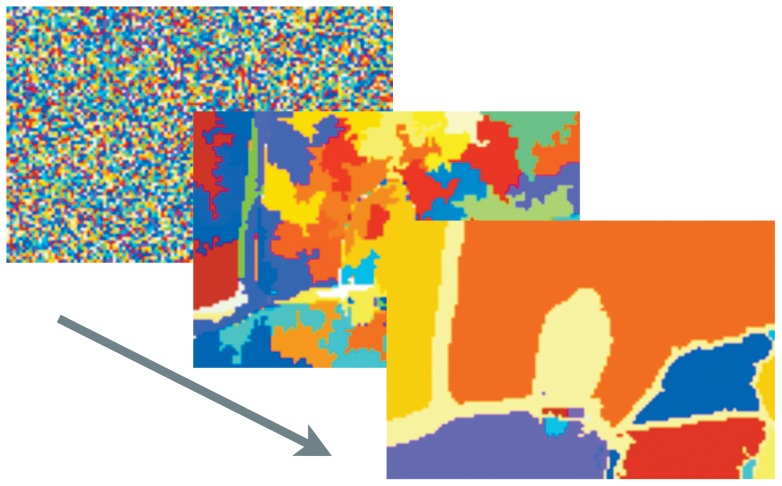
Visualization of the image segmentation process. Three out of about one hundred intermediate images are shown. In the first one on the left, every pixel is treated as a separate region; the last shows the final segmentation.

**Table 3:** Overview of segmentation results.

Rank	Score	Algorithm
0	0,79	Humans
1	0,68	Global Probability of Boundary [14]
2	0,66	xren [15]
3	0,65	Our method
4	0,64	Boosted Edge Learning [16]
…	…	…
12	0,41	Random

Results of various algorithms on the Berkeley segmentation dataset grayscale.

The last step describes the combination of the radial estimations into a density estimation of the whole space. It starts with the single radial estimation that is most likely. The next best of the remaining estimations is added iteratively and so forth. The selection ends when the increase of likelihood after adding another radial estimation stays below a threshold. In the example this process selected three radial estimations. The resulting final estimation is shown in diagram 3 of figure 3. We use the normalized leave-one-out likelihood as the selection criterion, see Section 3 of Appendix S1. The combination of multiple radial estimations into a single density is done by a mean operator (average of summation). The resulting Cepel model is:
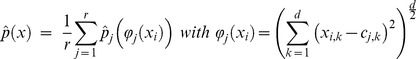
(3)



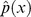
 *:* 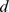 *-dimensional density estimation* 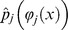 *: Density estimation of a one-dimensional projection* 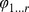 *: Radial projections around cluster centers* 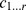


The example in figure 3 can be explained in more detail with equation (3). The selection prefers 1d-estimations located in new cluster centers, because they assign a high density to the points inside the cluster. Thereby the likelihood is increased significantly. This is not the case if a radial estimation is added that describes a cluster already included. It will increase the likelihood inside the cluster (approximately doubling it), but at the cost of decreasing the density everywhere else. In total the likelihood does not change considerably. This is the case after three iterations in fig. 3. The likelihood does not increase beyond the threshold by adding more estimations and therefore the process is terminated.

**Figure 11:**
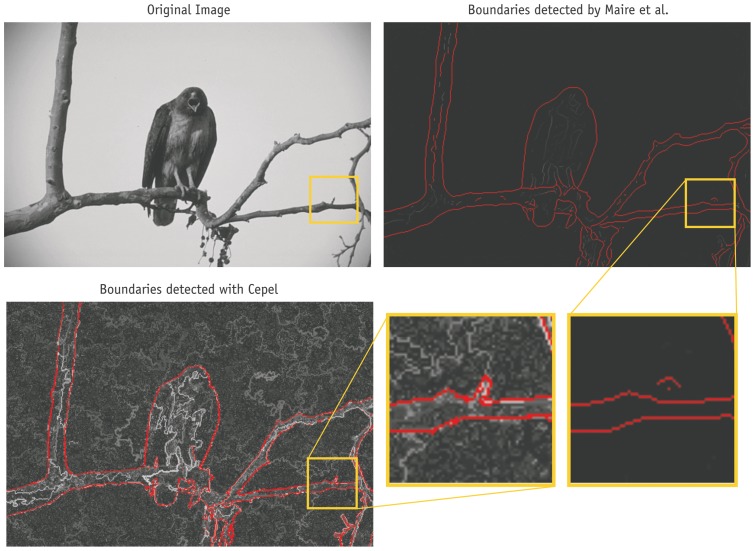
Comparison of segmentations of a filigree structure. Results of Maire et al. and the proposed method are compared at the example of a filigree structure. One threshold is highlighted in red for clarity.

**Figure 12:**
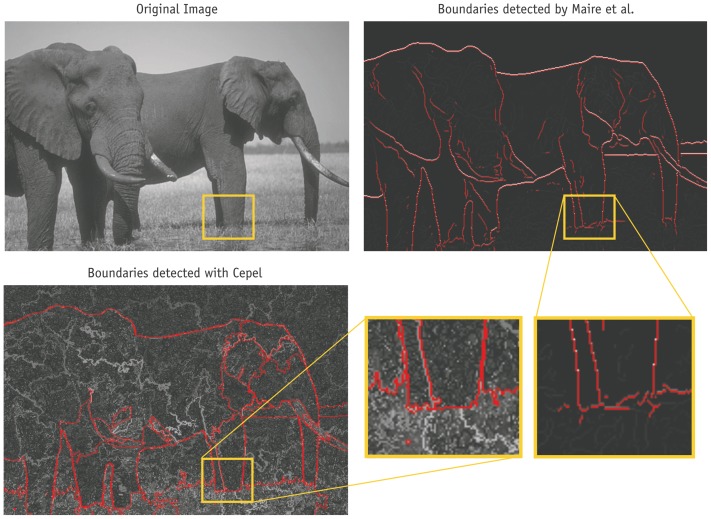
Comparison of a difficult patch. A second example of an image with a patch that is hard to segment.

This approach achieves superior results if the included assumptions about the problem are valid. In this case, the precision is nearly unaffected by the number of dimensions as all estimations are performed in a 1d-space. However, the estimation will be poor if the assumptions are violated, e.g. if clusters do not have a round shape, or if they are occluded in all projections and therefore their centers cannot be found.

A quantitative evaluation of the approach is given in figure 4. It compares the proposed method with a multidimensional Parzen window estimation [5]. The figure shows the normalized and scaled likelihood when the number of dimensions is increased from two to twelve. (Likelihood is scaled between zero and one. It is also normalized to compensate for the increase of support of additional dimensions, as described in Section 3 of Appendix S1.) Each additional dimension contains data with three clusters, like shown in figure 3. The bandwidth of Parzen window and the threshold of the proposed method are set using hold-out data. The figure illustrates that both methods improve at first, because each additional dimension contains independent information. With more than five dimensions the curse of dimensionality gets stronger and reduces the quality of the Parzen estimation significantly as expected. The reason is that Parzen window does not make additional assumptions about the data. The proposed method increases its precision continuously with additional dimensions of input space.

### Automatic Data Mining

Data mining is the process of discovering the most interesting characteristics of a large data set in order to understand it. Most data mining algorithms are semi-automatic, meaning that the user needs to pick a model and adjust parameters. This approach becomes less feasible as the size and complexity of data sets increase. This has lead to the recent trend towards fully automatic data mining, most prominently seen in Wolfram Alpha Pro (an online service by the creators of the software Mathematica, see [6]).

The goal of automatic data mining is to compile a report that summarizes the data without the requirement for user interaction. Therefore, the algorithms must be able to handle all kinds of data, like time series, n-dimensional samples, geographic information, log files and any kind of sensor data or measurements. Due to this generality, the methods are not able to yield the task-specific results of a specialized analysis. The advantage of automatic data mining is that it can give a quick overview of the data. It reveals relations that are unexpected and therefore might be missed by a specialized analysis.

In this paper, data mining is seen as a problem of density estimation. This is possible because various standard methods like linear regression, logarithmic or eigenvector analysis can be converted to 1d-decompositions. For example, a linear regression can be used for density estimation by projecting the data to its distance to the regression line. The corresponding equations for the linear regression and some other methods are given in table 1.

One advantage is that different methods become directly comparable, e.g. the results of Naive Bayes and a radial model can be compared. The criterion used for comparison is the likelihood of each model. The likelihood is a consistent criterion. Therefore, it is mathematically ensured that the correct model is selected if sufficient data is available. For example, the likelihood of the linear regression model will be maximal if the data contains a linear relation with additive noise. Again, the normalized leave-one-out likelihood is used, due to the reasons given in Section 3 of Appendix S1.

During the analysis, various decompositions are applied to a given data set. The most likely decomposition is selected and shown to the user. As each decomposition represents a certain linear or nonlinear correlation, this process is equivalent to selecting the predominant relations. Figure 5 illustrates the idea of calculating various models and selecting the best.

This approach is implemented in a software which we dub Cepel Inspect. It searches for 1d-decompositions of a data set and displays the most likely results in multiple diagrams. Currently, the analysis is limited to distributions of single features and relations between all possible pairs of features. Besides the continuous density estimation described, the software can handle discrete data as well. The whole approach is fully automatic and does not require any parameter setting or configuration. Therefore, it has proven useful for a quick initial analysis of unknown data. Figure 6 shows screenshots of the software. It is available online for evaluation [7].

Two examples of relations that can be revealed with this approach are given in the following. (Both examples can be reproduced easily by downloading the software and dragging the data file onto the program window. The two data sets used are “monitoring.csv” from [7] and “liver-disorders.csv” from [8].) Figure 7 shows an interesting correlation found in data from an oil refinery. The relation connects a continuously measured value (pressure in a pump) with a signal that is harder to measure but of high importance (normal/substandard performance of the pump). This insight has proven to be useful as it allows the cost-efficient identification of a pump with substandard performance, based on the measurement of its pressure.

The upper diagram displays an excerpt of the original time series. In its left half the performance of the pump is normal; on the right side it changed to substandard. By just looking at this diagram it could be concluded wrongly that both sides look similar and therefore there is no relation between pressure and performance. The lower diagram of figure 7 displays the same data, but this time as a distribution. The green curve shows that the pressure of pumps with substandard performance is often high or low (two maxima). In contrast, the blue curve indicates that pressure of normal pumps is mostly average (a single maximum). The difference between the curves is a clear indicator of a relation between pressure and performance.

The second example from an analysis is given in figure 8. Its an excerpt from a medical data set created to find a relation between liver disorders and five blood tests connected to alcohol consumption. Aside of finding the expected relation, the software also reveals correlations between different blood tests. The one shown in figure 8 is a linear relation between two blood tests (aspartate transaminase and gamma-glutamyl transferase). It could prove useful as it allows to skip one of the blood tests if the relation is strong enough to infer it from the second measurement. In the diagram the linear relation is indicated by the diagonal line which is the first eigenvector. The used 1d-decomposition projects the data onto both eigenvectors and multiplies the densities (as illustrated on the right of figure 2). The resulting density estimation is shown in light blue.

These examples demonstrate that density estimation by 1d-decomposition can be applied to practical data. The approach allows the extraction of valuable information in a fully automatic manner.

### Image Segmentation

A very different task that can also be interpreted as density estimation is image segmentation. It is the well-studied problem (see e.g. [9]–[10]) of dividing an image into visually homogenous regions. When interpreted as density estimation it translates to the question: how probable is it that two regions of arbitrary size and shape should be merged? A reliable answer to this question can be used to create a precise image segmentation. Mathematically, the answer is equivalent to estimating the posterior probability 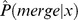. In the following, we use a vector 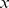 that consists of four features, as described in table 2. They are either used to estimate 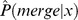 directly or for the intermediate probability 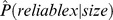.

The idea of the method is to divide the problem into two parts: 1) Estimation of 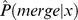 from the features brightness, texture and arrangement. This is done with a Naive Bayes model. 2) Increase the stability of this estimation with an intermediate probability 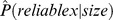.

The first step is illustrated in the left diagram of figure 9. The chart shows a 2d-space spanned by two (out of a total of three) features. The space is filled with data points that are calculated from random pairs of regions. If the regions are part of the same segment, the point is green; otherwise it is red. The curves above and on the left of the 2d-space are posterior probabilities of the data. They are created by projecting all red and green points on each axis. Then their densities are calculated using the proposed kernel estimator, resulting in two likelihood functions for each axis. The posterior is determined by combining both with Bayes theorem (see e.g. [11]). The prior probability required is calculated from the data, too.

Each posterior gives a partial description of the distribution of the data. Therefore, it helps to answer the stated question of how likely two regions are merged. For example, the feature on the horizontal axis is the difference of brightness of each region. When it is small, the probability of two regions belonging to the same segment is high, i.e. regions with similar brightness are more likely to belong together. The probability decreases with a larger difference of brightness. The same is true for the feature texture on the vertical axis, but this time the curve is flatter, meaning that texture is not as descriptive.

To calculate a final probability the information of features must be combined. In this case the features are chosen to be independent by design, i.e. it is assumed that the features brightness, texture and arrangement are independent. Therefore, their multiplication is optimal for calculating a combined probability, which means that the Naive Bayes approach is reasonable.

The second step of the method stems from a problem with this calculation. Sometimes features cannot be determined reliably. An example is texture, which cannot be accurately extracted from very small regions. Using such unreliable features would result in a distortion of the estimated probability. Therefore, another 1d-decomposition is performed for each of the three features. The right side of figure 9 illustrates this decomposition by the example of texture. The goal is to estimate the reliability of texture depending on the size of the region it is calculated from, which is 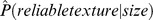. The posterior shows that texture is unreliable for regions with fewer than eight pixels, i.e. here the posterior is below 0.5. This estimation of reliability is calculated for each feature. It is used to include only reliable features into the calculation of 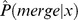. For example, if a region consists of seven pixels, only the posterior of brightness and shape are multiplied to calculate the final probability. For regions with three or less pixels, only brightness is used to estimate if it should be merged.

Combining both 1d-decompositions of figure 9 allows to estimate the probability that two regions are part of the same segment with high precision. The data required for estimation is created by analyzing random pairs of regions from images, for which a ground truth segmentation is known. The Berkeley image segmentation data set [12] is used for that. The whole segmentation model can be summarized in the following equations (with 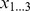 being the features brightness, texture and shape):
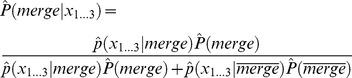
(4)
*with Naive Bayes*



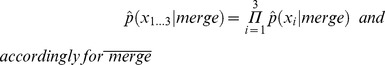
(5)
*including each* 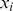 *only if*

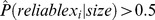
(6)


In the next step, the final probability 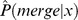 is used to perform the segmentation. This process is visualized in figure 10. It starts with an image, in which each pixel is treated as a separate region. The probability of all pairs of adjacent regions is calculated, resulting in (about) four probability values per pixel. Then an iterative merging begins. In each iteration the two regions with the highest probability are merged, creating a new region. The newly formed region has new features. Therefore, the probabilities of merging it with its neighbors are recalculated. The process terminates if all remaining regions are more likely to stay separate than to be merged. The following four steps summarize the process:

1. Calculate probabilities of merging each pair of adjacent regions.
2. Merge most likely pair of regions.
3. Recalculate probability of merged region.
4. Continue with step 2, until every probability is below 0.5.

The segmentation method was evaluated on 100 test images of the Berkeley benchmark data set. The approach achieved a score of 0.65, which is the third-best result published on the Berkeley website, see table 3. (We use the maximal F-measure as a scoring function. This is the standard score used in the Berkeley benchmark. It is calculated by comparing the boundary pixels of a method with the boundary defined by humans. This is done for different thresholds. The threshold, for which the result is maximal, is reported as the score, see [13].) In contrast to the two better performing methods (described in [14]–[15]), the features used are much simpler. Furthermore, only five out of 200 training images had to be used for estimation of the densities. Additional training does not increase precision considerably.

On average the proposed approach is less precise than two other methods. However, in some areas it has interesting advantages over the other segmentation approaches. Two examples are given in figure 11 and 12. (The images do not show the final segmentation as in figure 10 but the posterior probability of a boundary, i.e. the probability that two regions are not merged. This visualization shows more details and is the only form in which the results of Maire et al. are publicly available. The posterior is represented by gray values. Additionally, one threshold on the posterior is highlighted in red for clarity. The threshold is chosen manually so that the different methods are most comparable.) In the first figure a branch is highlighted as an example of a fine structure. Typical segmentation methods infer a boundary at a location from the pixels of two half discs around it, see [14]. For small structures like the branch, the half discs are too large to allow a precise segmentation, i.e. it is not possible that the branch is covered by one half disc while the other half contains background only. This results in an imprecision of the method of Maire et al. The algorithm is not able to segment the branch correctly, as shown in the highlighted image patch. In contrast, the proposed approach can extract very filigree structures, because it does not require a rigid area for calculation of features. Therefore, the branch is segmented with high precision.

Figure 12 focuses on the segmentation around the leg of an elephant. Finding a border here is difficult when taking only the local neighborhood into account. Therefore, the method of Maire et al. does not find a continuous border below the leg. The proposed approach succeeds in this case, because of the order in which it merges regions. It combines pairs of regions with high probability first, meaning that homogenous areas are merged before complex regions. Difficult parts like the leg are segmented late in the process when features can be calculated from larger regions with higher precision. In this example, the whole leg and large parts of the ground are segments before deciding that the features of both regions are quite dissimilar. Postponing hard decisions until more information from other areas is available is advantageous and increases the overall precision of segmentation.

## Discussion

In this paper, we have introduced a new framework for density estimation. It is based on 1d-decomposition – the projection of data onto 1d-spaces, in which densities are estimated and combined back to a multidimensional model. The framework allows a fully automatic and fast computation of 1d-estimations because 1d-spaces have unique properties for optimization.

Three examples demonstrate the wide range of applications for which a 1d-decomposition is possible. They show how 1d-projections can be used to incorporate assumptions and thereby increase the precision of estimation. The clustering and image segmentation example focus on adjusting the framework to specific prior knowledge about a task. The automatic data mining application illustrates the capacity of the method to function without task-specific knowledge. This is achieved by testing for generic relations that appear commonly in data, like clusters of points and independence or linearity of features.

We would like to encourage the reader to test the application by oneself. The software can be downloaded at www.cepel.de together with some data sets. Reproducing the results as shown e.g. in figures 6 and 7 requires only a few moments.

One line of future work will concentrate on extending the image segmentation. Its precision would benefit greatly from more advanced texture features and color information. The flexibility of the framework ensures that this additional information could be included efficiently. The increase of precision opens up another application: the model could be used not only to create segments but also to describe them. This allows to recognize segments in different images. Thereby, the method could function as a novel kind of interest point descriptors for segments.

The data mining application could be extended by including additional models. In particular, they could cover non-linear correlations between more than two features. Even very complex relations can be discovered this way. For example, there is no reason why the model used for image segmentation could not be found automatically. The limiting factor is the requirement of a decomposition into 1d-distributions. However, as shown in this paper, for a diverse set of problems a 1d-decomposition is possible. Therefore, we expect many more areas of application in the future.

## Supporting Information

Appendix S1 **The appendix consists of three sections covering: 1d-density estimation, runtime remarks, and model selection.**
(DOC)
