# Supplementary material for: A Projection and Density Estimation Method for Knowledge Discovery
Source: PLoS One. 2012 Oct 1;7(10):e44495. doi: 10.1371/journal.pone.0044495 (PMC3462203; doi:10.1371/journal.pone.0044495)
Supplement: Appendix S1 — The appendix consists of three sections covering: 1d-density estimation, runtime remarks, and model selection. (DOC) [file pone.0044495.s001.doc]

## S1 Appendix - Supporting Information

## 1. 1d-Density Estimation

The proposed method relies on an automatic 1d-density estimation. This estimation must be precise for data from any continuous probability distribution, independent of its form and the amount of data available. It cannot require any parameters (except for precision-performance tradeoffs), because that would prevent it from being automatic. Its only input is the 1d-data from which it has to estimate the most likely density. In the following, an adaptive kernel estimator with parameter selection is described that achieves this goal.

### Kernel Estimator

Kernel density estimators were developed to estimate the continuous distribution of a function with unknown form [5]. In their most popular variant they put a Gaussian function on each data point. The sum of all Gaussians is the estimated density. In this context, the Gaussian functions are called kernels and their standard deviation is the bandwidth. The choice of the bandwidth is crucial for the precision of the estimation.

The most simple way for setting the bandwidths is to select the same value for all kernels. Unfortunately, in this way it is not possible to select a bandwidth that is equally optimal for denser and sparser areas of the distribution. It will either oversmooth dense regions or undersmooth areas of low density. This results in artifacts in the estimation, like isolated kernels instead of smooth transitions in spare regions.

Kernel estimators with an adaptive bandwidth are a solution for this problem. They adjust the bandwidth depending on the local distribution of data points. Typically, the bandwidth is varied in proportion to the distance to the point, which is the -nearest in the data, see [17]. As intended, this results in high bandwidths in areas of low density; denser regions will be smoothed less.


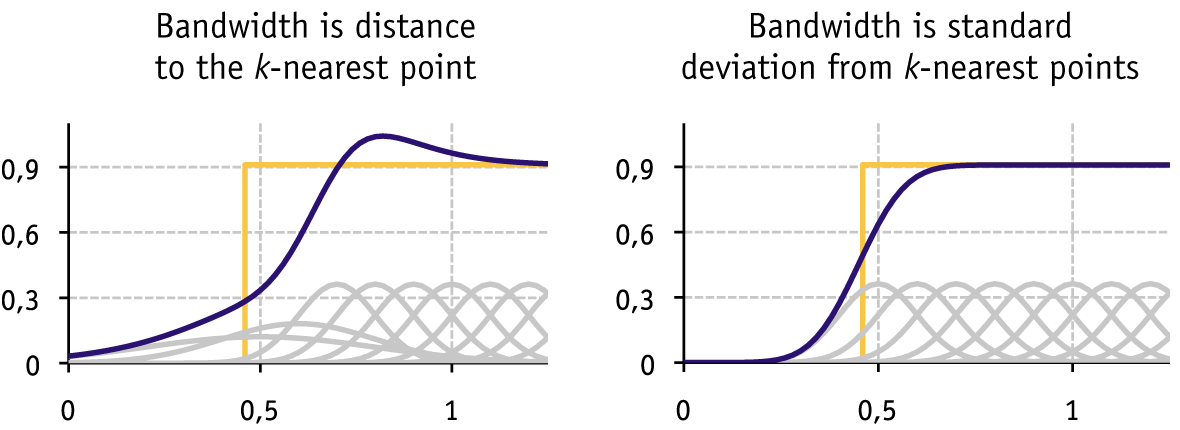


Fig. S1: The typical kernel estimator with adaptive bandwidth suffers from artifacts at sharp changes in density (left). The chosen approach is more precise (right). The true density (yellow) is approximated by the estimation (blue), which is a summation of Gaussians (grey).

Although this adaptive strategy can yield a higher precision, it is not accurate at sharp changes in density. This is illustrated on the left diagram of figure S1. By changing the bandwidth continuously the estimator creates a bump, similar to the Gibbs phenomenon, see [18]. A kernel estimator without this drawback was proposed by [2]. It adapts bandwidths depending on the expected standard deviation of the set of -nearest points. It is constant near sharp density changes, making the estimation more precise, as shown in the right diagram of figure S1.

Another source of inaccuracy of adaptive bandwidths appears when estimating real data. In practice, data is often not a set but a multiset, i.e. a point can occur multiple times. Neglecting this fact results in various problems, like Dirac delta impulses at points that appear more often than the of the adaptive bandwidth. Distinguishing between sets and multisets while calculating the adaptive bandwidth resolves this issue. The resulting equation for the adaptive kernel estimator used in this paper is:

(7)

with and (8)

: Data point with index from multiset of data with size

: Multiset of k-nearest neighbors closest to

: Number of data point in multiset

: Number of data points in set of (without repetitions)

,: Empirical standard deviation and mean of

### Parameter Selection

To create an automatic kernel estimator, its free parameter must be chosen automatically. Simple heuristics for doing this depend only on the size of the data, like , see [4]. Therefore, e.g., a smooth distribution and a “wiggly” one of equal size will be assigned the same . This results in a lower precision than achievable by taking the distribution of data into account.

More elaborate methods evaluate the data with different parameters and select the setting that maximizes some criterion, see e.g. [19-20]. We use the normalized leave-one-out likelihood function for this, see Appendix C. Calculating it for every possible in equation *(7)* and selecting the maximum, leads to the required automatic kernel density estimator:

(9)

## 2. Runtime Remarks

Kernel density estimation is an inherently slow procedure. Combining it with leave-one-out cross validation, another complex method, makes its direct application infeasible. However, in the 1d-setting analyzed in this paper and in combination with various optimization methods, the computation can be reduced severely to a practical runtime. The idea behind each optimization strategy is given in the following.

### -Nearest Neighbors Calculation

A major reason for the slow computation of kernel estimations of multidimensional data is the search for the -nearest neighbors, see e.g. [21-22]. A naive implementation would require the calculation of distances between all points. However, in one dimension this is simplified greatly: after sorting the data, the closest point to a given value can be identified by binary search; its nearest neighbors are found by traversing the data points with nearby indices. The calculation of -nearest neighbors is only that efficient in one dimension, because here the order of points is related closely to their distances. For multidimensional data this relation does not hold.

### Kernelscope

Another significant decrease of runtime is based on the observation that far away data points have very limited influence on the density. For example, neglecting a Gaussian with a distance of six times its standard deviation, results in an error of only of its probability mass. Therefore, only the Gaussian functions in the neighborhood have to be considered when calculating the density of a point. We call this approximation kernelscope, because it limits the influence of a kernel to its local scope.

Calculation of a density at one point with equation *(7)* requires summation of -functions. The kernelscope allows to approximate this independent of . The strength of approximation is adjusted with parameter ; smaller values result in a higher performance. The default value used is . The modified version of equation *(7)* with kernelscope is ( is index of data point closest to ):

with (10)

### Sample Scale Space

The kernelscope will reduce runtime as long as bandwidths are small. It cannot speedup the calculation of wide Gaussians, because they influence distant densities considerably. A wide Gaussian that spans over a large part of data will be estimated from many data points, i.e. mean and standard deviation in equations *(8)* will be calculated from a large number of points. Calculating these parameters from a proper subset will not change a wide Gaussian significantly, but it reduces runtime.


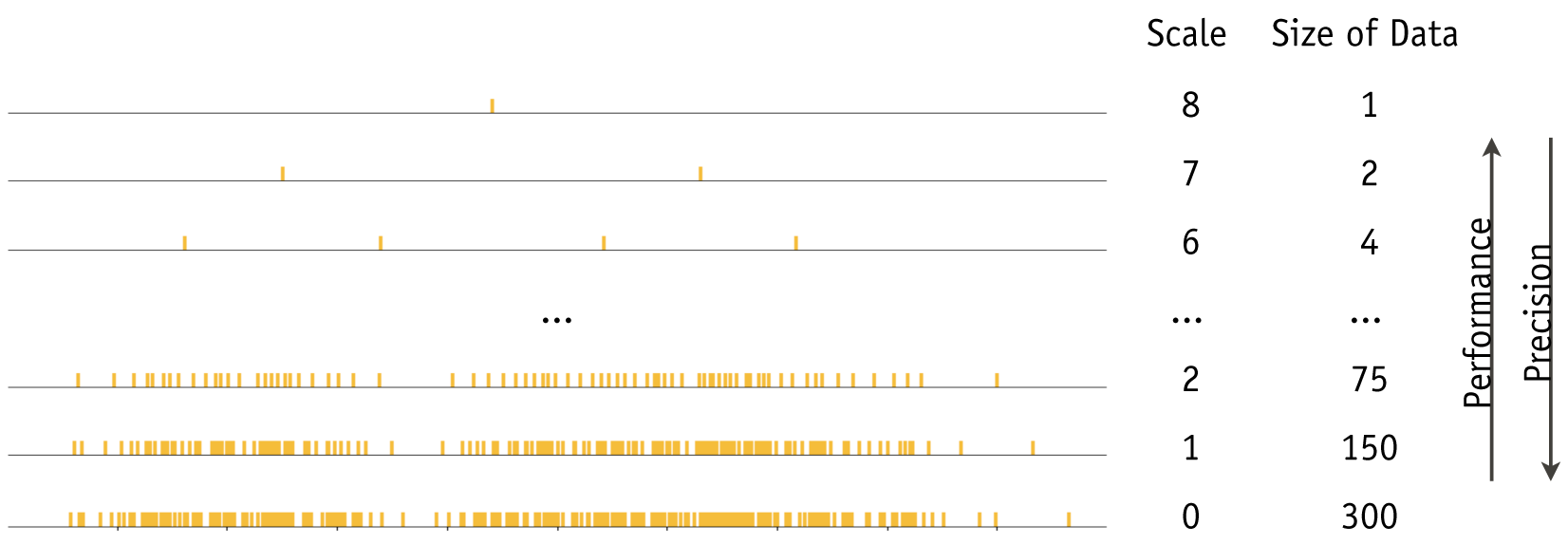


Fig. S2: Example of the sample scale space with eight scales and 300 data points.

This insight is exploited in an approximation technique called sample scale space. It works similarly to a scale space in computer vision. The idea is to perform calculations that comprise a large area on a high scale, which contains only a fraction of data. Operations that cover only a limited region are performed on a low scale with high precision. This way, the sample scale space reduces runtime for large bandwidths. In combination with the kernelscope that speeds up small bandwidths, the overall performance of kernel estimation is high enough for practical applications.

An example of a sample scale space is given in figure S2. Each scale contains half of the data of its predecessor. The full data set is in scale zero. Scale one contains every other point of sorted data of scale zero; scale two every fourth point and so forth. The sample scale space is used to approximate calculations that involve a large number of points. Instead of calculating with points on the full data set the calculation is performed on a higher scale with a smaller number of points . For example, the estimation of the mean of points on scale zero is approximated by the mean of points on scale . As scale contains only of points of the full data set, is calculated from with equation:

(11)

Selecting a suitable scale for a calculation requires a trade-off between the fast computation of a high scale and the precision of a low scale. The larger the higher the scale that allows a precise estimation. Calculations with small depend on few points only. Therefore, they cannot be approximated efficiently. This trade-off is achieved by selecting the scale with equation *(12)*. It contains the parameter that adjusts the strength of approximation. The smaller the stronger the approximation at the cost of precision. For all calculations in this paper it is set to .

when (12)

## 3. Model Selection

Model selection is performed multiple times throughout this paper, e.g. in the introductory example for choosing cluster centers, for data mining to select decompositions and in kernel estimation for picking parameter . In all cases the models at choice are different probability density estimations. Therefore, the likelihood function is applicable, which is a consistent criterion that selects the correct model if sufficient data is available. However, in practice two problems arise which should be discussed here.

The first issue regards overfitting. It results, e.g., in falsely selecting the kernel with smallest when applying likelihood directly. It can be handled by using different subsets of data during model selection and training of the model. This is done in the kernel estimator by means of *leave-one-out likelihood* cross validation.

The second problem appears when selecting between models with different support. For example, during data mining models with one and two dimensions are compared, see figure 5. The likelihood would probably select the one-dimensional model, as its density is distributed over a smaller space and therefore is larger. Hence, a *normalization of likelihood* with respect to support is required. The volume of support is approximated with the minimal hypercube containing all data. It is used to normalize the likelihood as shown in equation *(13)*. This way, the likelihood of models with different support can be compared directly. In practice this equation is calculated as a sum of log-factors to avoid precision issues. The *normalized leave-one-out likelihood criterion* used in this paper is defined as:

with (13)

: Data set consisting of points

: Model which is a probability density estimation

: Leave-one-out estimation given without , required for kernel estimation in eq. (9)

: Volume of hypercube containing all data

: Element of -dimensional data point with index

References (Appendix)

1. Loftsgaarden DO, Quesenberry CP (1965) A Nonparametric Estimate of a Multivariate Density Function. Ann of Math. Statistics.
2. Gibbs JW (1898) Fourier Series. Nature.
3. Loader CR (1999) Bandwidth selection: classical or plug-in? Ann of Statistics.
4. Wasserman L (2007) All of Nonparametric Statistics. 3rd printing. Springer.
5. Gray AG, Moore AW (2003) Nonparametric Density Estimation: Toward Computational Tractability. SIAM Data Mining.
6. de Berg M, van Kreveld M, Overmars M, Schwarzkopf O (2008) Computational Geometry - Algorithms and Applications. 3nd edition. Springer.
